# Baseline incidence of intussusception in early childhood before rotavirus vaccine introduction, the Netherlands, January 2008 to December 2012

**DOI:** 10.2807/1560-7917.ES.2017.22.25.30556

**Published:** 2017-06-22

**Authors:** Kartini Gadroen, Jeanet M Kemmeren, Patricia CJ Bruijning-Verhagen, Sabine MJM Straus, Daniel Weibel, Hester E de Melker, Miriam CJM Sturkenboom

**Affiliations:** 1 Department of Medical Informatics, Erasmus MC, Rotterdam, the Netherlands; 2 Medicines Evaluation Board (CBG-MEB), Utrecht, the Netherlands; 3 Center for Infectious Disease Control, National Institute for Public Health and the Environment (RIVM), Bilthoven, the Netherlands; 4 Julius Center for Health Sciences and Primary Care, University Medical Center Utrecht, Utrecht, the Netherlands

**Keywords:** Retrospective cohort study, rotavirus vaccine safety, intussusception incidence rate, primary healthcare data, IPCI, hospital data, LBZ

## Abstract

Intussusception is a rare, potentially life-threatening condition in early childhood. It gained attention due to an unexpected association with the first rotavirus vaccine, RotaShield, which was subsequently withdrawn from the market. Across Europe, broad variations in intussusception incidence rates have been reported. This study provides a first estimate of intussusception incidence in young children in the Netherlands from 1 January 2008 to 31 December 2012, which could be used for future rotavirus safety monitoring. Our estimates are based on two different sources: electronic medical records from the primary healthcare database (IPCI), as well as administrative data from the Dutch hospital register (LBZ). The results from our study indicate a low rate of intussusception. Overall incidence rate in children < 36 months of age was 21.2 per 100,000 person-years (95% confidence interval (CI): 12.5–34.3) based on primary healthcare data and 22.6 per 100,000 person-years (95% CI: 20.9–24.4) based on hospital administrative data. The estimates suggest the upper and lower bound of the expected number of cases.

## Introduction

Rotavirus infections are a leading cause of severe diarrhoeal illness in infants and young children [1]. As demonstrated by a number of studies, rotavirus vaccines are effective in preventing severe diarrhoeal illness caused by certain rotavirus serotypes [2,3]. In 1999 however, the first rotavirus vaccine, RotaShield (Wyeth Laboratories, Inc., Pennsylvania, United States), was voluntarily withdrawn from the market due to an unexpected association with intussusception [4]. Intussusception is a serious condition that can be described as the invagination of a proximal segment of the bowel into the distal bowel. If left untreated, the blood flow can become compromised, leading to bowel infarction and perforation. In 2006, two second generation rotavirus vaccines, Rotarix (GlaxoSmithKline Biologicals, Rixensart, Belgium) and RotaTeq (MSD vaccines, Lyon, France), were approved for marketing in Europe. In 2009, the World Health Organization Strategic Advisory Group of Experts (SAGE) recommended the use of rotavirus vaccines in all national immunisation programs, and by 2016, 11 countries of the European Union had included rotavirus vaccination in their national vaccination programme [5]. Although large-scale pre-licensure clinical trials did not identify an increased risk for intussusception, post-licensure data suggested a small increase in risk of intussusception that was closely linked to the age of vaccination after rotavirus vaccination with both licensed vaccines [6-11].

The aetiology of primary intussusception in young children remains unclear. Intussusception is most common between 5 and 7 months of age [12]. Approximately 60% to 75% of children diagnosed with intussusception are younger than 1 year of age, and approximately 80% to 90% are younger than 2 years of age. Most episodes occur in otherwise healthy children with a male to female predominance of ca 3:2 [13].

Ultrasonography is the method of choice to detect intussusception. Ultrasound-guided reduction using hydrostatic or pneumatic pressure by enema is the treatment of choice. Surgical treatment is indicated when ultrasound-guided reduction is incomplete or in case perforation is suspected.

Sentiments towards the importance of vaccination in general are positive overall but confidence in vaccine safety is less positive, particularly in the European region [14]. In the Netherlands, where the impact of rotavirus vaccine is considered modest [15], concerns about vaccine safety may lead to vaccine hesitancy and decreased vaccination coverage for vaccine-preventable diseases in general [16]. Knowledge of the background incidence rates of possible adverse events is a crucial part of assessing possible vaccine safety concerns. It allows for a rapid observed vs expected analysis and helps to distinguish legitimate safety concerns from events that are temporally associated with but not necessarily caused by vaccination [17]. In the case of rotavirus vaccination, it is important to know the background incidence of intussusception. Studies in Europe have reported incidence rates of intussusception between 24.2 and 60.4 per 100,000 person-years [18-23] and show a decline over time [19,22]. Methods used to estimate these incidence rates differ in age of source population, length of study period, and detection and validation of cases.

To date, the use of rotavirus vaccines in the Dutch population can be considered negligible. Rotavirus vaccines are not included in the Dutch national vaccination programme, are not recommended for routine use and are not reimbursed by the health insurance. To support future rotavirus vaccine safety surveillance in the event that rotavirus vaccine would be introduced in the Dutch national vaccination programme, this study aims to calculate the baseline incidence rates of intussusception in the Netherlands.

## Methods

### Data sources

For this study two different data sources that capture a partially overlapping source population were used: an administrative hospital discharge database and a primary healthcare database. Administrative hospital discharge data were obtained from the Dutch hospital register, Landelijke Basisregistratie Ziekenhuiszorg (LBZ). Hospitals and university medical centres in the Netherlands have a legal and statutory obligation to collect and provide electronic administrative data to the LBZ database on a monthly basis. The LBZ database covers more than 80% of the total Dutch population of 17 million people. It contains anonymised data on hospital admissions, outpatient consultation and emergency department visits including medical diagnoses, as well as patient-specific data such as age and sex [24]. Coding of discharge diagnoses is performed by participating hospitals according to the International Classification of Diseases, Ninth Revision (ICD-9) [25]. A validation study showed high accuracy of coding and concluded that the discharge data are generally of high quality [26].

The second source was a primary healthcare database, the Integrated Primary Care Information (IPCI) database. IPCI is a longitudinal observational database created specifically for pharmaco-epidemiological studies [27]. It contains anonymised data, including notes from computer-based medical records of around 600 general practitioners (GPs) located in the Netherlands. IPCI contains information on more than 1.1 million patients from over 200 participating GP practices. The age and sex distribution of the population is representative for the Netherlands. In the Dutch healthcare system, the GP acts as a gatekeeper for all medical care. It is estimated that more than 75% of the Dutch population in the age group 0–3 years will visit their GP at least once per year [28]. In the event intussusception is suspected, the patient will be referred to hospital for confirmation and treatment. Following consultation at the hospital, it is standard practice to forward the details of the consultation and the outcome to the GP. In the rare event a patient by-passes the GP, the hospital will also forward the details of the consultation and the outcome to the patient’s GP. Therefore, patients’ medical records at GP practices are likely to contain all relevant medical information.

### Study design and population

Cases were retrieved differently from the two data sources. From the LBZ hospital discharge database, all cases with a primary or secondary discharge diagnosis of intussusception (ICD-9 CM code 560.0) in children aged between 0 and 36 months (i.e. children aged 0–35 months) at admission were retrieved for the period from 1 January 2008 to 31 December 2012. Cases with a secondary diagnosis of intussusception were reviewed by a paediatrician to determine whether the combined set of ICD codes for the admission was compatible with a new occurrence of intussusception, taking comorbidities, patient age and primary discharge diagnosis into account. Possible duplicate reports because of patient transfers were identified based on sex, birthdate and date of diagnosis, and were excluded.

Based on the data captured by IPCI, we constructed a dynamic cohort. Initially, we attempted to assess the incidence rate over a period of 10 years, from 1 January 2003 to 31 December 2012. However, the addition of many new practices to the IPCI database in 2007 caused the observation time to vary considerably over the course of the 10-year study period and we subsequently restricted the analysis to a period of 5 years, from 1 January 2008 to 31 December 2012. This yielded a more stable population (Table 1).

**Table 1:** Incidence rate of intussusception per 100,000 person-years based on validated cases from the Integrated Primary Care Information (IPCI) database, the Netherlands, January 2003–December 2012

Year	Cases (n)	Person time (person-years)	Incidence rate per 100,000 person-years	95% CI
2003	0	2,226	NA	NA
2004	1	2,323	43.0	3.9–200.7
2005	0	4,549	NA	NA
2006	0	978	NA	NA
2007	0	1,495	NA	NA
2008	2	4,045	49.4	9.9–158.5
2009	2	8,435	23.7	4.7–76.0
2010	4	14,087	28.4	9.5–67.5
2011	2	19,326	10.3	2.1–33.2
2012	5	24,649	20.3	7.7–44.5

We only selected children who were born during the study period and who had contributed longitudinal data to the IPCI database from birth onwards. Follow-up started from birth and continued until the date the patient became a case, the patient reached the age of 36 months, the patient died, the patient was transferred out of the GP’s practice, the date of last data collection from the general practice or the end of the study period was reached, whichever date came first. In the IPCI database, cases were identified by automated scanning of keywords in GPs’ notes in the medical records. The complete medical records of all potential cases were reviewed by a medical doctor for details alluding to additional diagnostic procedures or received treatment. An identified case was considered a true case if the medical journal of that particular patient contained results from ultrasound examination confirming the diagnosis and/or details regarding receiving treatment specific for intussusception such hydrostatic reduction. Cases with an actual diagnosis of intussusception were subsequently classified by level of evidence using the web-based Automated Brighton Collaboration Case definition tool (ABC-tool) for intussusception. Level one corresponds to the highest level of diagnostic evidence and level three corresponds to the lowest [29]. We subsequently compared LBZ and IPCI data for those years where comparable data were available.

### Analysis

Age-specific incidence rates were calculated from data in each of the two data sources using the number of intussusception cases from that source as a numerator and the study population as denominator. Since dominator data are not available in the LBZ database, approximate denominators based on national population data from Statistics Netherlands (CBS) were used, assuming that 80% of the population would be covered in the LBZ database [30]. In the IPCI database, the underlying study cohort could be well defined in terms of the number of person-years of follow-up of patients. Incidence rates of intussusception were calculated by dividing the number of incident intussusception cases by the total number of person-years. Incidence rates were calculated by calendar year, age category and sex. Confidence intervals (95% CI) for each estimate were based on the Poisson distribution.

## Results

### Landelijke Basisregistratie Ziekenhuiszorg (LBZ) database

**Figure 1:**
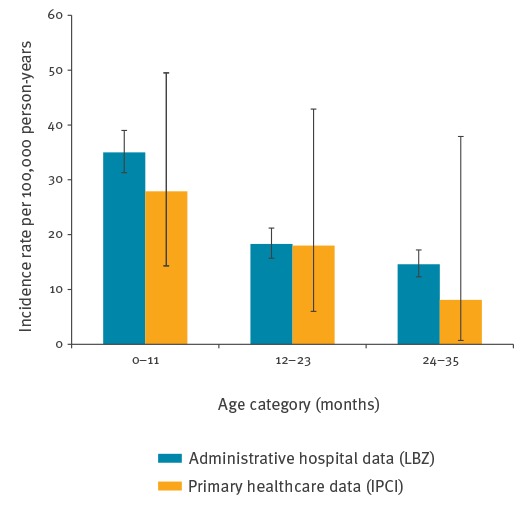
Intussusception incidence rate in children < 36 months of age per 100,000 person-years by age and data source, the Netherlands, 1 January 2008–31 December 2012

IPCI: Integrated Primary Care Information database; LBZ: Landelijke Basisregistratie Ziekenhuiszorg database.

In the LBZ database, 705 potential cases of intussusception were identified during the study period and 166 duplicate cases were excluded. Based on the remaining 539 identified cases of intussusception, the overall crude incidence rate over the study period from 1 January 2008 to 31 December 2012 was 22.6 per 100,000 person-years (95% confidence interval (CI): 20.9–24.4). Figure 1 shows the age-specific incidence rates; the highest rate was observed in children < 12 months of age.

Vertical lines represent the 95% confidence intervals.

**Figure 2:**
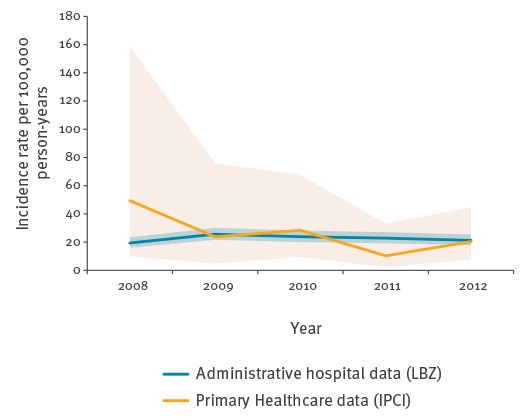
Intussusception incidence rate in children < 36 months of age per 100,000 person-years by year and data source, the Netherlands, 1 January 2008–31 December 2012

IPCI: Integrated Primary Care Information database; LBZ: Landelijke Basisregistratie Ziekenhuiszorg database.

The incidence rate remained constant over time (Figure 2).

Shaded areas represent the 95% confidence intervals.

**Figure 3:**
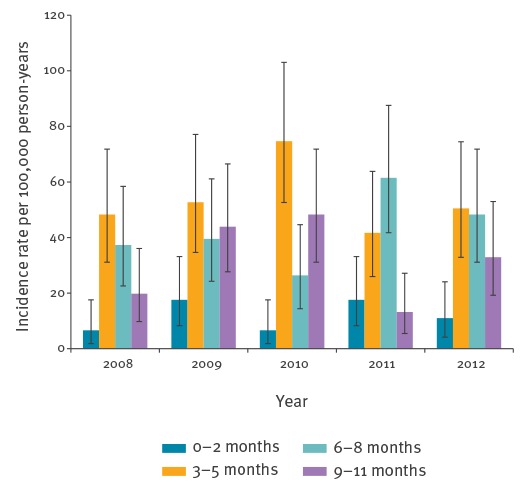
Intussusception incidence rate in children < 12 months of age per 100,000 person-years by age category and year based on non-validated cases from the LBZ database, the Netherlands, 1 January 2008–31 December 2012

The incidence rate in children < 12 months of age varies considerably by age, but as is expected, the data show that is it more common after the age of three months (Figure 3).

LBZ: Landelijke Basisregistratie Ziekenhuiszorg.

The LBZ database contains administrative hospital data.

Vertical lines represent the 95% confidence intervals.

### Integrated Primary Care Information (IPCI) database

From the IPCI database, 155,880 children were included in the initial study cohort (Table 2). Within this population, 131 potential cases were detected after a sensitive search for indicators of intussusception in narratives. We subsequently restricted age-specific analysis to the period from 1 January 2008 to 31 December 2012, which yielded a more stable population. In the period from 1 January 2008 to 31 December 2012, the population comprised of 144,617 children. Following manual validation by a medical doctor, 15 cases (14 definite, 1 possible) were classified as incident intussusception (Table 1). When using the ABC-tool, all cases were classified as having insufficient information to meet the Brighton Collaboration case definition of intussusception. This was because none of the cases included any information regarding one of the exclusion criteria: absence of surgical evidence for an alternative diagnosis. Based on the number of cases validated by a medical doctor, the crude incidence during the period 1 January 2008 to 31 December 2012 was 21.2 per 100,000 person-years (95% CI: 12.5–34.3). The incidence was higher in boys than in girls, and was highest in the lowest age category, subsequently decreasing with age (Table 3). Results per age category are provided in Figure 1, and overall incidence per calendar year in Figure 2.

**TABLE 2:** Study cohort details for investigation of intussusception incidence rates using the IPCI database, the Netherlands, 1 January 2008–31 December 2012

Study cohort	Time period	
	1 Jan 2003–31 Dec 2012	1 Jan 2008–31 Dec 2012
Study population (n)	155,880	144,617
Person time of follow up (person-years)	82,113	70,542
Number of intussusception cases (n)	16	15

IPCI: Integrated Primary Care Information.

The IPCI database contains general practitioner medical records.

**Table 3:** Intussusception incidence rates in children < 36 months of age per 100,000 person-years based on validated cases from the IPCI database, the Netherlands, January 2003–December 2012

Sex and age	Time period			
	1 Jan 2003–31 Dec 2012		1 Jan 2008–31 Dec 2012	
	Incidence rate per 100,000 person-years	95% CI	Incidence rate per 100,000 person-years	95% CI
Overall	20.2	12–32	21.3	12.5–34.3
Sex				
Male	26.7	14.2–46.2	27.7	14.2–49.2
Female	12.7	4.8–27.9	14.6	5.5–32
Age				
0–11 months	24.6	12.6–43.7	27.9	14.3–49.5
12–23 months	19.8	7.5–43.3	18	6.0–42.9
24–35 months	6.9	0.6–32	8.1	0.7–37.9

IPCI: Integrated Primary Care Information.

The IPCI database contains general practitioner medical records.

## Discussion

This study showed that background incidence rates of intussusception can be estimated using routinely collected healthcare data. The intussusception incidence rate in children < 12 months of age is 27.9 per 100,000 person-years (95% CI: 14.3–49.5) based on cases from the primary healthcare data that were validated, and 35.0 per 100,000 person-years (95% CI: 31.3–39.0) based on the non-validated hospital data. These estimates are on the lower end of published incidences across Europe. Consistent with previous research [19,22,23,31], the incidence rate in boys was higher than in girls, and was highest in the youngest age group.

When comparing the intussusception incidence rates derived from the IPCI primary healthcare database with those derived from the LBZ hospital database, the results from the IPCI database are validated but less precise, and possibly an underestimation. From the hospital database, we were able to derive precise incidence rates for smaller age categories. However, the coding could not be validated and the incidence rate may possibly be an overestimation. A declining trend over time was not evident. The true intussusception rate is likely to be in between the estimates derived from the primary healthcare database and the hospital database. The advantage of this dual approach is that interpreting the occurrence of future cases after vaccination may be done with or without validation, and we have provided an assessment of the impact thereof.

The advantage of using a primary healthcare, GP-based database such as the IPCI was that case detection did not depend on the validity of coding as free text keyword searches, rather than codes, were used. The availability of medical notes in free text provided a rich source of information, enabling case ascertainment. Although it would be interesting to know the composition of the participating practices, we were not able to identify any evidence that would suggest that the composition of the GP practice is a risk factor for intussusception. Therefore, we consider it unlikely that our estimate is biased by the composition of the GP practices. As a denominator, we were able to use accrued time since birth. However, intussusception is a condition typically diagnosed in a hospital setting. Although it is considered standard practice to forward all relevant hospital patient data to the GP, it cannot be ruled out that some hospital diagnoses were not communicated to GPs or were substantially delayed in terms of being reported back. Since the number of ascertained cases was small, we could only calculate the incidence for age categories of one year and the confidence intervals are rather large.

The majority of first-time rotavirus infections usually occur in infancy. In high income countries, 65% occurs in infants < 1 year of age [32]. Therefore, it is recommended that rotavirus vaccination be administered before the age of 6 months. However, the reported incidence of intussusception varies substantially by age during the first 6 months of life [23,33]. Age-specific incidence rates in months or even weeks of age would be very useful in terms of informing rotavirus vaccine safety policy; however, this would require a much larger cohort.

Because the administrative hospital database covers a larger population than the primary healthcare one, we were able to derive more precise incidence rates and incidence rates for smaller age categories. Incidence rates for small age categories is particular valuable information in the context of rotavirus vaccine safety surveillance since studies suggest that the risk of intussusception caused by rotavirus vaccine is primarily in the first week after the first dose, administered in early childhood [6-11]. However, denominator data are not readily available and approximate denominators from population census data have to be used. In addition, case detection in this particular hospital database depends on the accuracy of coding and could not be validated. Published positive predictive values of ICD-9 codes for intussusception range from 75% to 81%, and are even lower when including outpatient department data [34-36]. Therefore, the hospital database estimates may be an overestimation of the true incidence. Moreover, in contrast to the primary healthcare database, the intussusception cases derived from the hospital database may contain cases of transient intussusception, recurrent intussusception and/or suspected intussusception. In order to further investigate the quality of the estimates derived from the LBZ database, a validation study could be considered. In the future, if hospital data are to be used for intussusception surveillance, distinguishing suspected intussusception cases and transient intussusception cases from true intussusception cases might be of added value.
